# Association of C‐Reactive Protein‐Triglyceride Glucose Index and TyG Index With Prehypertension and Hypertension

**DOI:** 10.1002/iid3.70486

**Published:** 2026-07-14

**Authors:** Shijie Yang, Yuqing Zhang, Zhanyang Zhou, Mingying Xu, Long Feng, Xiaotian Zu

**Affiliations:** ^1^ AnZhen Hospital of the Capital University of Medical Sciences Beijing China; ^2^ Department of Cardiology, Fu Wai Hospital Chinese Academy of Medical Sciences and Peking Union Medical College Beijing China

**Keywords:** anthropometric indicator, C‐reactive protein‐triglyceride glucose index, hypertension, prehypertension, Triglyceride‐glucose index

## Abstract

This study aimed to investigate the associations of the C‐reactive protein‐triglyceride glucose index (CTI) and the triglyceride‐glucose (TyG) index with prehypertension and hypertension in a Chinese population aged 45 years and older. Based on blood pressure diagnostic criteria, 8389 eligible participants were categorized into three groups. In the multivariate logistic regression analysis, after adjusting for potential confounders (Model 2), individuals in the highest quartiles (Q4) of both indices exhibited a significantly increased risk of hypertension compared to those in the lowest quartiles (Q1). Specifically, the adjusted odds ratios (ORs) for Q4 were 1.592 (95% CI: 1.354–1.872) for the TyG index and 1.588 (95% CI: 1.356–1.860) for CTI. A similar independent association was observed for prehypertension. Notably, the CTI demonstrated an enhanced responsiveness in identifying hypertension at lower exposure levels, maintaining a significant association in the second quartile (Q2 OR: 1.201, 95% CI: 1.034–1.395). Furthermore, RCS analysis revealed a more pronounced non‐linear dose‐response relationship for CTI (P‐non‐linear < 0.001) than for TyG (P‐non‐linear = 0.045). Both CTI and the TyG index are independent indicators of prehypertension and hypertension in middle‐aged and elderly Chinese adults. Although their standalone discriminatory power is modest (AUC ≈ 0.59), CTI may serve as a supplementary and cost‐effective monitor for the early risk stratification of hypertension, given its responsiveness to non‐linear risk transitions.

## 1 Introduction

Hypertension stands as a paramount risk factor for cardiovascular disease (CVD). Over the past several decades, a substantial escalation in its prevalence has solidified hypertension as a formidable contributor to the global health burden, a trend that is especially acute in low‐ and middle‐income countries [1]. Amidst the rapid expansion of the global obesity epidemic over the past three decades, the profound impact of adiposity on the development of hypertension (HTN) has gained widespread recognition [2]. With a staggering prevalence of roughly 27.5%, hypertension affects an estimated 245 million adults in China, representing a significant proportion of the nation's cardiovascular disease burden [3]. Substantial body of clinical and epidemiological evidence demonstrates that insulin resistance (IR) is intricately linked to the pathogenesis of hypertension [4, 5, 6]. The co‐existence of IR and hypertension synergistically escalates the risk of developing CVD and type 2 diabetes mellitus (T2DM) [7]. The HIEC technique is the recognized gold standard for IR assessment but is impractical for widespread use due to its invasive nature [8]. Meanwhile, indirect measures like HOMA‐IR are hampered by the necessity of insulin assays and suboptimal reproducibility in repeated testing [9, 10]. Since its introduction in 2008 as a dependable indicator of IR, the TyG index has been extensively utilized in clinical research [11]. A growing body of literature indicates that the TyG index is significantly associated with the progression of atherosclerosis and hypertension, serving as a potent predictor for the prognosis of CVD [15].

Furthermore, chronic inflammation is recognized as a pivotal risk factor for the development of hypertension [12]. It can markedly elevate the incidence of hypertension by accelerating atherosclerosis, impairing vascular endothelial function, and promoting thrombosis [13, 14]. As a non‐specific inflammatory mediator, C‐reactive protein (CRP) is intimately linked to hypertension risk and has emerged as a promising biomarker for its clinical assessment [11, 15]. Therefore, developing a comprehensive index that integrates both IR and systemic inflammation is crucial for elucidating the complex interplay underlying hypertension, rather than relying solely on single‐dimensional metabolic markers. The CRP‐triglyceride glucose index (CTI), initially proposed by Ruan et al. to integrate systemic inflammation and IR, has gained significant traction in clinical research [16]. The CTI transitions from a single‐dimensional marker to an integrated “inflammatory‐metabolic” index. This allows it to capture the compounding, cross‐dimensional vascular damage that single surrogate markers might underestimate. While CTI has demonstrated robust predictive capacity for cardiovascular events and mortality [17, 18] across diverse populations, its specific association with hypertension and prehypertension—particularly across different glycemic profiles—remains inadequately explored.

## 2 Methods

### 2.1 Study Population

**Figure 1:**
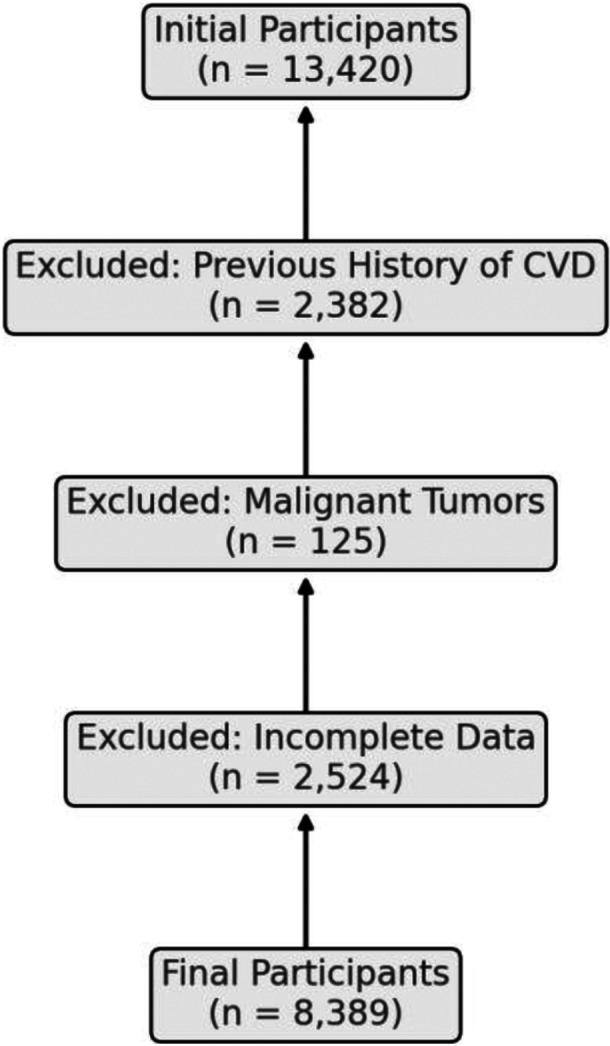
Participant selection flow chart.

CHARLS is a nationally representative longitudinal survey of the middle‐aged and older population (≥ 45 years) conducted by the National School of Development at Peking University. Detailed information on the study population has been reported in other publications statistical analysis [19]. The CHARLS nationwide baseline survey was initiated in 2011–2012, followed by subsequent waves in 2013 (Wave 2), 2015 (Wave 3), 2018 (Wave 4), and 2020 (Wave 5). Blood samples were collected during the baseline and Wave 3 surveys, involving 11,847 and 13,420 participants, respectively. The present study utilized Wave 3 data, focusing on individuals aged 45 years and older with complete records of fasting blood glucose (FBG) and triglycerides (TG). Exclusion criteria were: (1) a documented history of CVD, including coronary heart disease, stroke (ischemic/hemorrhagic), transient ischemic attack, peripheral vascular disease, valvular heart disease, heart failure, and aneurysm; (2) malignant tumors; and (3) incomplete data. Ultimately, 8389 eligible participants were included in the final analysis (Figure 1).

### 2.2 Clinical Data and Biochemical Indicators

Study participants underwent a comprehensive assessment comprising a standardized questionnaire, a detailed physical examination, and venous blood sampling. The questionnaire was employed to collect demographic information and clinical characteristics, including age, sex, smoking status, medical history, and current medication use. Anthropometric measurements were conducted using standardized procedures. Height and weight were measured, and body mass index (BMI) was calculated as the body weight in kilograms divided by the square of height in meters. Waist circumference (WC) was determined by measuring the midline between the lower rib margin and the iliac crest while the participant stood upright. Blood pressure was measured by trained staff with an upper arm medical electronic sphygmomanometer after at least 5 min of rest. A total of three measurements were taken with an interval of 1 min, and the average value of the last two measurements was recorded as office blood pressure. Primary prevention included antihypertensive treatments, lipid‐lowering treatments, and hypoglycaemic treatments. Laboratory examination contained fast blood glucose (FBG), total cholesterol (TC), triglyceride (TG), CRP, and low‐density lipoprotein cholesterol (LDL‐c) [20].

### 2.3 Definitions

TyG index = Ln[TG (mg/dL) × FPG (mg/dL)/2];

CTI = 0.412 × Ln (CRP [mg/L]) + Ln (TG [mg/dl] × FPG [mg/dl])/2

Normotension was defined as systolic BP (SBP) < 120 mmHg and diastolic BP (DBP) < 80 mmHg. Prehypertension was defined as 120 ≤ SBP < 140 mmHg and/or 80 ≤ DBP < 90 mmHg. Hypertension was defined as systolic blood pressure (SBP) ≥ 140 mmHg and/or diastolic blood pressure (DBP) ≥ 90 mmHg [21]. Individuals with a hypertensive history and meanwhile taking antihypertensive medication should also be diagnosed as hypertension [22].

### 2.4 Statistical Analysis

The distribution of continuous variables was assessed using the Kolmogorov–Smirnov test. Normally distributed variables were expressed as mean ± standard deviation (SD), while skewed variables were presented as median and interquartile range (IQR). Categorical variables were reported as frequencies and percentages (%). Differences between groups were evaluated using one‐way analysis of variance (ANOVA) for continuous data and the chi‐square test for categorical data. A *p*‐value of less than 0.05 was considered statistically significant.

Spearman's correlation analysis was performed to explore the associations between CTI, TyG, and other clinical parameters. To evaluate the independent associations of CTI and TyG with prehypertension and hypertension, multivariate logistic regression models were constructed. Both indices were analyzed as continuous variables and as categorical variables divided into quartiles, with the lowest quartile (Q1) serving as the reference group. Two adjustment models were utilized: Model 1 was unadjusted, and Model 2 was adjusted for age, sex, smoking status, TC, LDL‐c, and diabetes. To further characterize the potential non‐linear dose‐response relationships between CTI, TyG, and the risk of hypertension, restricted cubic spline (RCS) analysis was performed with four knots. The discriminatory power of CTI and TyG for identifying patients with hypertension or prehypertension was evaluated using receiver operating characteristic (ROC) curves. The area under the curve (AUC) and its 95% confidence interval (CI) were calculated, and the DeLong method was employed to compare the AUCs of different parameters. The optimal cut‐off values were determined by the maximum Youden index. Statistical analyzes were performed using STATA version 17.0 software (StataCorp, College Station, Texas) and R software (version 4.4.1; R Foundation for Statistical Computing, Vienna, Austria). Two‐sided *p* < 0.05 was considered statistically significant.

## 3 Results

### 3.1 Characteristics of the Study Population

Based on blood pressure status, 8389 eligible participants were categorized into three groups: normotension (*n* = 3148), prehypertension (*n* = 3025), and hypertension (*n* = 2216). The study population had a median age of 61 years, with men (*n* = 4024) comprising 47.9% of the total. As summarized in Table 1, baseline characteristics—including age, sex, smoking, alcohol consumption, HR, BMI, blood pressure, lipid profiles, and uric acid—differed significantly across the three groups (*p* < 0.001). Notably, individuals in the hypertension group exhibited markedly higher BMI, WC, TC, TG, and LDL‐c levels compared to those in the prehypertension and normotension groups.

**Table 1:** Characteristics of the study population.

Variables	All participants (*n* = 8389)	Normotension (*n* = 3148)	Prehypertension (*n* = 3025)	Hypertension (*n* = 2216)	*p*‐value
Age (years)	61 (53–67)	58 (52–65)	61 (54–67)	63 (57–70)	< 0.001
Gender (men)	4024 (47.9%)	1373 (43.6%)	1511 (50.0%)	1140 (51.4%)	< 0.001
Current smoking	2447 (29.2%)	917 (29.1%)	880 (29.1%)	650 (29.3%)	< 0.001
Current drinking	3063 (36.5%)	1072 (34.1%)	1146 (37.8%)	845 (38.2%)	< 0.001
Diabetes (%)	1508 (18.0)	399 (12.7)	588 (19.4)	521 (23.5)	< 0.001
BMI (kg/m^2^)	23.4 (21.1–25.9)	22.5 (20.4–24.8)	23.7 (21.5–26.2)	24.4 (21.9‐26.8)	< 0.001
WC (cm)	85.5 (78.5–92.5)	82.6 (76.0–89.4)	86.5 (79.9–93.3)	88.6 (81.2–95.8)	< 0.001
TC (mg/dL)	181.5 (160.2–205.4)	177.4 (156.8‐200.8)	182.2 (161.4–206.2)	185.7 (163.3–211.4)	< 0.001
LDL‐C (mg/dL)	101.2 (83.0–119.3)	98.1 (81.1–116.6)	102.3 (83.4–120.1)	103.1 (85.3–123.2)	< 0.001
CRP (mg/L)	1.4 (0.7–2.6)	1.2 (0.6–2.3)	1.4 (0.8–2.6)	1.6 (0.9–2.9)	< 0.001
TG (mg/dL)	112.4 (81.4–167.3)	102.6 (77.0–151.3)	115.9 (84.1–175.2)	123.0 (87.6–182.3)	< 0.001
UA (mg/dL)	4.8 (3.9–5.7)	4.6 (3.8–5.5)	4.9 (4.0–5.8)	5.0 (4.0–6.0)	< 0.001
Scr (mg/dL)	0.76 (0.66–0.89)	0.75 (0.65–0.88)	0.77 (0.66–0.90)	0.78 (0.67–0.92)	< 0.001
SBP (mmHg)	125 (113–139)	110 (104–115)	128 (123.5–133)	149.0 (143.5–157.5)	< 0.001
DBP (mmHg)	74.0 (67.0–82.0)	66.5 (61.5–71.0)	76.5 (71.0–81.0)	86.5 (79.5–93.5)	< 0.001
HR	73 (66.5–80.5)	73.0 (67.0–79.5)	73.0 (66.5–80.5)	73.5 (66.5–81.5)	< 0.001
TyG	8.6 (8.2–9.1)	8.5 (8.2–8.9)	8.6 (8.3–9.1)	8.7 (8.3–9.2)	< 0.001
CTI	8.8 (8.2–9.4)	8.6 (8.1–9.2)	8.8 (8.3–9.5)	8.9 (8.5–9.6)	< 0.001

Abbreviations: BMI, body mass index; CTI, C‐reactive protein‐triglyceride glucose index; CRP,C‐Reactive Protein; DBP, diastolic blood pressure; HR, heart rate; LDL‐C, low‐density lipoprotein cholesterol; SBP, systolic blood pressure; Scr, serum creatinine; TC, total cholesterol; TG, triglycerides; TyG, triglyceride glucose index; WC, waist circumference; UA, uric acid.

### 3.2 Correlation Analysis

Spearman's correlation analysis (Table 2) demonstrated that both TyG and CTI were significantly and positively correlated with HR, BMI, WC, SBP, DBP, TC, LDL‐c, and UA (all *p* < 0.001). Conversely, a significant negative correlation was observed between Scr and both TyG (*r* = −0.052, *p* < 0.001) and CTI (*r* = −0.020, *p* < 0.001). Notably, while TyG exhibited a weak negative correlation with age (*r* = −0.058, *p* < 0.001), no statistically significant association was found between age and CTI (*r* = 0.002, *p* = 0.85). Overall, CTI generally displayed slightly higher correlation coefficients (r) with SBP, DBP, HR, and WC compared to the TyG index.

**Table 2:** Spearman correlation between TyG and CTI and clinical parameters.

Parameters	TyG		CTI	
	*r*	*p* value	*r*	*p* value
Age	−0.058	< 0.001	0.002*	0.85
SBP	0.149	< 0.001	0.161	< 0.001
DBP	0.153	< 0.001	0.158	< 0.001
HR	0.171	< 0.001	0.201	< 0.001
BMI	0.373	< 0.001	0.371	< 0.001
WC	0.390	< 0.001	0.403	< 0.001
Scr	−0.052	< 0.001	−0.020	< 0.001
TC	0.306	< 0.001	0.260	< 0.001
LDL ‐ c	0.095	< 0.001	0.076	< 0.001
UA	0.193	< 0.001	0.228	< 0.001

* Means *p* > 0.05.

### 3.3 Association of TyG Index and CTI With Hypertension

Table 3 presents the multivariate logistic regression analysis evaluating the associations of TyG and CTI with the prevalence of hypertension. Both indices were categorized into quartiles, with the lowest quartile (Q1) serving as the reference. In both Model 1 and Model 2, a consistent and significant positive trend was observed between increasing quartiles of TyG and CTI and the risk of hypertension (*p* < 0.001). After adjusting for potential confounders in Model 2 (including age, sex, smoking, TC, LDL‐C, and diabetes), participants in the highest quartile (Q4) of both indices exhibited a significantly elevated risk of hypertension. Specifically, the adjusted odds ratios (ORs) for Q4 versus Q1 were 1.592 (95% CI: 1.354–1.872) for TyG and 1.588 (95% CI: 1.356–1.860) for CTI. CTI maintained a statistically significant association with hypertension across all quartiles (Q2 OR: 1.201, 95% CI: 1.034–1.395; Q3 OR: 1.487, 95% CI: 1.283–1.723), suggesting that CTI may be a more sensitive indicator for hypertension risk at lower levels of exposure.

**Table 3:** Multivariate logistic regression analysis of TyG, CTI, and related parameters for the prevalence of hypertension.

Variables	Quartile 1	Quartile 2	Quartile 3	Quartile 4	*p* for trend
TyG	*n* = 2101	*n *= 2095	*n* = 2096	*n* = 2097	
Model 1	Reference	1.169 (1.011–1.351)	1.442 (1.252–1.661)	1.751 (1.524–2.012)	< 0.05
Model 2	Reference	1.155 (0.995–1.340)	1.403 (1.210–1.627)	1.592 (1.354–1.872)	< 0.001
CTI	*n* = 2098	*n* = 2097	*n* = 2097	*n* = 2097	
Model 1	Reference	1.252 (1.082–1.449)	1.615 (1.401–1.862)	1.869 (1.624–2.150)	< 0.001
Model 2	Reference	1.201 (1.034–1.395)	1.487 (1.283–1.723)	1.588 (1.356–1.860)	< 0.001

*Note:* Model 1: adjusted for no confounding factors; Model 2: adjusted for age, gender, smoking habitats, TC, LDL‐C, diabetes; *Means *p* > 0.05.

### 3.4 Association of TyG Index and CTI With Prehypertension

As indicated in Table 4, a consistent upward trend in the prevalence of prehypertension was observed with increasing quartiles of TyG and CTI in both Model 1 and Model 2 (P for trend < 0.001). Compared to the hypertension group, the magnitude of the association for these parameters was relatively attenuated. After adjusting for potential confounding factors in Model 2, both TyG and CTI remained significantly and independently associated with prehypertension. In the fully adjusted model, TyG exhibited an OR of 1.398 (95% CI: 1.208–1.617) for the highest versus the lowest quartile. Notably, CTI demonstrated a slightly higher associative strength in the second quartile (OR: 1.266, 95% CI: 1.113–1.440) compared to TyG (OR: 1.148, 95% CI: 1.009–1.306), although both reached their peak ORs in Q4. These results suggest that while both indices are robust indicators of prehypertension, CTI may offer an early risk‐signaling benefit in identifying individuals at the earliest stages of blood pressure elevation.

**Table 4:** Multivariate logistic regression analysis of TyG and CTI for the prevalence of prehypertension.

Variables	Quartile 1	Quartile 2	Quartile 3	Quartile 4	*p* for trend
TyG	*n* = 2101	*n* = 2095	*n* = 2096	*n* = 2097	
Model 1	Reference	1.143 (1.006–1.299)	1.166 (1.026–1.325)	1.354 (1.193–1.536)	< 0.05
Model 2	Reference	1.148 (1.009–1.306)	1.171 (1.027–1.335)	1.398 (1.208–1.617)	< 0.001
CTI	*n* = 2098	*n* = 2097	*n* = 2097	*n* = 2097	
Model 1	Reference	1.275 (1.122–1.449)	1.285 (1.131–1.461)	1.391 (1.225–1.580)	< 0.001
Model 2	Reference	1.266 (1.113–1.440)	1.269 (1.114–1.446)	1.378 (1.195–1.590)	< 0.001

*Note:* Model 1: adjusted for no confounding factors; Model 2: adjusted for age, gender, smoking habitats, TC, LDL‐C, diabetes; *Means *p* > 0.005.

### 3.5 Association of CTI and TyG Index With the Prevalence of Hypertension Visualized by Restricted Cubic Splines

**Figure 2:**
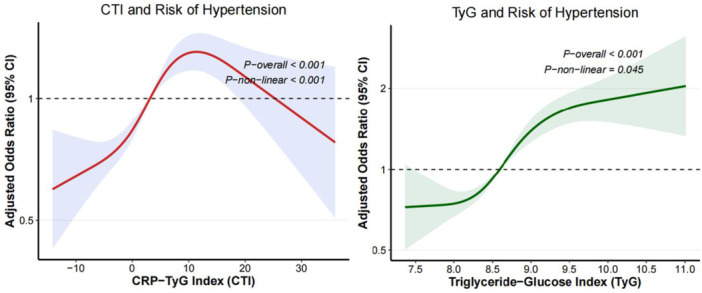
Restricted cubic spline (RCS) plots illustrating the dose‐response associations of CTI and TyG index with the risk of hypertension.

Analysis of the RCS models revealed distinct dose‐response relationships for CTI and TyG index with the risk of hypertension (Figure 2). While both indices exhibited strong overall associations with hypertension (P‐overall < 0.001), the CTI demonstrated a more pronounced and complex non‐linear relationship (P‐non‐linear < 0.001) compared to the TyG index (P‐non‐linear = 0.045). As shown in the plots, the risk of hypertension escalated sharply as CTI levels increased, reaching a peak at approximately CTI = 12, followed by a slight attenuation at extremely high levels. In contrast, the TyG index showed a more sustained, sigmoid‐like upward trend after exceeding a threshold of approximately 8.5. These findings suggest that incorporating CTI significantly enhances the sensitivity for identifying the non‐linear risk transition of hypertension, particularly at moderate exposure levels.

### 3.6 Predictive Value of TyG and CTI for Hypertension

**Figure 3:**
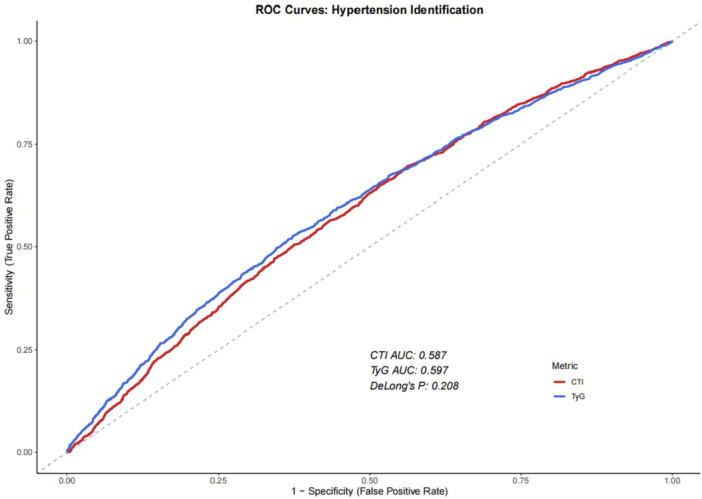
Receiver operative characteristic curves for identifying hypertension by TyG index and CTI.

According to Figure 3, both indices demonstrated statistically significant predictive value for the presence of hypertension (*p* < 0.001). The AUC for the TyG index was 0.597 (95% CI: 0.582–0.613), while the AUC for CTI was 0.587 (95% CI: 0.571–0.602). To further compare the predictive performance of these two markers, the DeLong test was performed. The results revealed no statistically significant difference between the AUC of TyG and that of CTI (*p* = 0.208), suggesting that both indices possess comparable diagnostic accuracy in identifying hypertensive patients within this middle‐aged and elderly Chinese population. Based on the Youden index, the optimal cut‐off values for identifying hypertension were determined to be 8.7 for the TyG index and 0.83 for CTI. These cut‐off points may serve as practical thresholds for clinical risk stratification.

## 4 Discussion

To the best of the authors’ knowledge, this is a large‐scale cross‐sectional study investigating the associations of the CTI and the TyG index with prehypertension and hypertension in a Chinese population aged 45 years and older. The primary findings of this study are as follows: The main findings included: (1) The prevalence of hypertension in this study population was 26.4%. Participants in the hypertension group presented with a more adverse metabolic profile, characterized by significantly higher levels of BMI, WC, TC, TG, and LDL‐c compared to the prehypertension and normotension groups; (2) Spearman's correlation analysis demonstrated that both TyG and CTI were positively correlated with key cardiovascular risk factors, including HR, BMI, WC, SBP, DBP, and lipid profiles. Notably, CTI displayed stronger correlation coefficients with blood pressure parameters (SBP and DBP) and HR than the TyG index; (3) RCS analysis revealed a more pronounced and complex non‐linear relationship for CTI (P‐non‐linear < 0.001) compared to TyG. While both indices showed comparable overall diagnostic accuracy in identifying hypertension via ROC analysis (TyG AUC: 0.597; CTI AUC: 0.587; *p* = 0.208), the integration of inflammation into the CTI index provides a more comprehensive reflection of the non‐linear risk transition.

The TyG index was originally developed as a validated surrogate marker for IR. A wealth of evidence has demonstrated that IR is intrinsically linked to a spectrum of pathological conditions, including diabetes, obesity, coronary artery disease, and coronary artery calcification. These metabolic derangements are largely driven by compensatory hyperinsulinemia and its subsequent systemic effects [23, 24, 25, 26, 27]; An analysis of UK biobank data found elevated baseline TyG index was associated with a higher risk of CVD after adjustment for the well‐established CVD risk factors [28]; Another prospective study demonstrated that among individuals with central obesity, those in the higher TyG quartiles (Q3: 8.52–8.95; Q4: 8.95–12.14) exhibited a significantly increased risk of hypertension compared to those in the lowest quartile (Q1: 4.96–8.18) [29]. The pivotal role of IR in hypertension development is well‐recognized, despite the existence of partially undefined mechanistic pathways. Hyperinsulinemia, a hallmark of IR, is believed to trigger a cascade of events—including the stimulation of the sympathetic nervous system and the renin‐angiotensin‐aldosterone system—that collectively exacerbate vascular resistance and blood pressure levels [30, 31]; hyperglycemia elevates the osmotic pressure in the extracellular space relative to the intracellular environment, which restore osmotic equilibrium, water shifts into the bloodstream, thereby increasing both the circulating blood volume and the blood pressure within the vessels [32]. Multiple studies have explored the association between the TyG index and related parameters with hypertension, a study involving 47,808 individuals highlighted that the Triglyceride‐glucose (TyG) index exhibited a stronger association with hypertension compared to simple glycemic and lipid indicators, establishing it as an independent predictor of hypertension [33]; A prospective and observational clinical study suggested the elevated TyG index was a potential marker of adverse prognosis in patients with CHD and hypertension [34]; However, Wu et al. noted that while the TyG index significantly associated with the progression of arterial stiffness in hypertensive individuals, this association was not observed in the prehypertensive population [35]. Another longitudinal study was reported that the TyG index to be a robust indicator for incident hypertension, and Cox regression analyses from the study indicated that a higher TyG index is associated with an increased risk of developing hypertension subsequently [36].

Developed by Ruan et al., the CTI serves as a multifaceted indicator by synthesizing the inflammatory biomarker CRP with the metabolic index TyG [16]. While the individual components of the CTI—namely IR and systemic inflammation—have been extensively linked to adverse cardiovascular outcomes like CVD, their combined impact on blood pressure regulation warrants closer examination [37]. For instance, although the TyG index is a recognized predictor of hypertension and atherosclerosis across diverse clinical cohorts, including middle‐aged and hypertensive individuals, it primarily captures the metabolic dimension of vascular risk [38]. Recent evidence emphasizes that utilizing integrated, multi‐dimensional biomarker panels provides a significantly more comprehensive reflection of systemic microvascular and metabolic cross‐talk than relying on single‐dimensional indices alone, establishing a clear link between compounded inflammatory pathways and adverse clinical severity [39]. By mathematically bridging IR and systemic inflammation into a single coupled index, the CTI functions within this same holistic framework, offering a more nuanced capturing of the early pathological transitions that precede overt vascular remodeling. Similarly, the causal relationship between CRP and stroke risk, as evidenced by Mendelian randomization, underscores the independent role of inflammation in vascular damage [40]. Given the recent findings by Cui et al. regarding the mutual mediation and co‐exposure effects of the TyG index and CRP on cardiovascular health, the CTI may offer a more comprehensive reflection of the inflammatory‐metabolic interplay [41]. Establishing the association between CTI and hypertension is therefore a logical progression in refining risk stratification for the elderly population, potentially offering an early risk‐signaling benefit over single‐dimensional markers like the TyG index alone.

Several limitations associated with this study should be acknowledged. First, although we utilized office blood pressure measurements, the lack of ambulatory blood pressure monitoring (ABPM) data—which could identify masked or white‐coat hypertension—remains a limitation. Secondly, this study did not account for longitudinal changes in glycemic status or inflammatory markers over time. Future research should incorporate dynamic assessments of the CTI and metabolic status to more comprehensively evaluate their temporal relationship with blood pressure progression. Future research directions will focus on establishing multicenter prospective longitudinal cohorts equipped with ABPM data. This will allow us to map the precise temporal trajectories of the CTI in relation to blood pressure progression and the development of masked or white‐coat hypertension. Thirdly, we did not adjust for baseline BMI, WC, serum uric acid, or primary prevention medications in our final models. Given the strong correlations observed between these metabolic parameters and the components of CTI/TyG (as shown in Table 2), this approach was intentionally chosen to prevent severe multicollinearity and model over‐adjustment. However, the possibility of residual confounding from unmeasured lifestyle factors or pharmaceutical therapies cannot be entirely ruled out. Lastly, as this study exclusively focused on middle‐aged and elderly Chinese individuals, the generalizability of our findings to younger cohorts or other ethnic populations may be limited. Future prospective studies are needed to validate the predictive and associative value of CTI in diverse clinical environments and populations. Furthermore, interventional trials are warranted to determine whether therapeutic modification of the CTI can effectively reduce the risk of hypertension and its related cardiovascular complications.

## 5 Conclusions

The TyG index and CTI exhibited significant and independent associations with hypertension in the population over 45 years old. While the standalone discriminative ability of both markers is modest, the CTI demonstrates an enhanced responsiveness in identifying individuals at the earliest stages of blood pressure elevation. This suggests that the CTI may serve as a valuable ancillary tool for early cardiovascular risk stratification rather than a definitive diagnostic test.

## Author Contributions

**Shijie Yang:** conceptualization, writing – original draft, methodology, writing – review and editing, formal analysis, software, data curation, resources. **Yuqing Zhang:** methodology, supervision, data curation. **Zhanyang Zhou:** methodology. **Mingying Xu:** methodology. **Long Feng:** methodology. **Xiaotian Zu:** conceptualization, methodology, writing – review and editing, supervision.

## Declaration of Generative AI and AI‐Assisted Technologies in the Writing Process

The authors declare that no generative AI or AI‐assisted software was employed to conduct data analysis, interpret clinical findings, or draft the core medical hypotheses and concepts presented in this manuscript.

## Funding

The authors have nothing to report.

## Ethics Statement

This study utilized data from the CHARLS, which obtained ethical approval from the Institutional Review Board at Peking University (IRB00001052‐11015). The current study involved secondary analysis of publicly available, de‐identified data, and as such, further ethical approval was not required.

## Consent

Informed consent was obtained from all participants by the CHARLS. As this study involved secondary analysis of publicly available data, no additional patient consent was required.

## Conflicts of Interest

The authors declare no conflicts of interest.

## Data Availability

The data that support the findings of this study were obtained from CHALRS under license and are not publicly available. Data are available from the authors upon reasonable request and with permission from CHALRS.
